# Towards an accessible, centralised, searchable database for AI courses in Europe: the Artificial Intelligence in Medical Imaging and Radiation Oncology Education (AIMIROE) project

**DOI:** 10.1186/s41747-026-00745-8

**Published:** 2026-05-29

**Authors:** Robin Decoster, Hendrik Erenstein, Jacob Menzinga, Patrizia Cornacchione, Altino Cunha, Elona Dybeli, Nejc Mekis, Mark McEntee, Karoliina Paalimäki-Paakki, Helle Precht, Tugba Akinci D’Antonoli, Renato Cuocolo, Merel Huisman, Michail E. Klontzas, Elmar Kotter, Daniel Pinto dos Santos, Erik Ranschaert, Peter van Ooijen, Nikolaos Stogiannos, Christina Malamateniou

**Affiliations:** 1 Department of Medical Imaging and Radiation Therapy, Odisee University of Applied Sciences, Brussels, Belgium; 2 European Federation of Radiographer Societies, Cumieira, Portugal; 3 Department of Medical Imaging and Radiation Therapy, Hanze University of Applied Sciences, Groningen, The Netherlands; 4 Department of Radiotherapy, University of Groningen, University Medical Centre Groningen, Groningen, The Netherlands; 5 Research Group Healthy Ageing, Allied Health Care and Nursing, Hanze University of Applied Sciences, Groningen, The Netherlands; 6 Policlinico Universitario Agostino Gemelli, Agostino Gemelli, Italy; 7 Università Cattolica del Sacro Cuore, Rome, Italy; 8 Department of Medical Technical Specialties, Faculty of Medical Technical Sciences, University of Elbasan “Aleksander Xhuvani”, Elbasan, Albania; 9 Faculty of Health Sciences, University of Ljubljana, Ljubljana, Slovenia; 10 Discipline of Medical Imaging and Radiation Therapy, University College Cork, Cork, Ireland; 11 Department of Radiography and Radiation Therapy, Oulu University of Applied Sciences, Oulu, Finland; 12 Health Sciences Research Centre, UCL University College, 5230 Odense M, Denmark; 13 Department of Radiology, Lillebaelt Hospital, University Hospitals of Southern Denmark, Kolding, 6000 Kolding, Denmark; 14 Institute of Regional Health Research, University of Southern Denmark, Odense, Denmark; 15 Department of Diagnostic and Interventional Neuroradiology, University Hospital Basel, Basel, Switzerland; 16 Department of Pediatric Radiology, University Children’s Hospital Basel, Basel, Switzerland; 17 European Society of Medical Imaging Informatics, Vienna, Austria; 18 Department of Medicine, Surgery and Dentistry, University of Salerno, Baronissi, Italy; 19 Department of Radiology and Nuclear Medicine, Radboud University Medical Center, Nijmegen, The Netherlands; 20 Artificial Intelligence and Translational Imaging (ATI) Laboratory, Department of Radiology, Medical School, University of Crete, Voutes, Heraklion, Crete Greece; 21 Division of Radiology, Department for Clinical Science Intervention and Technology (CLINTEC), Karolinska Institutet, Stockholm, Sweden; 22 Computational Biomedicine Laboratory, Institute of Computer Science, Foundation for Research and Technology (FORTH), Heraklion, Crete Greece; 23 Department of Diagnostic and Interventional Radiology, Medical Center—University of Freiburg, Faculty of Medicine, University of Freiburg, Freiburg, Germany; 24 Department of Radiology, University Medical Center Mainz, Mainz, Germany; 25 Faculty of Medicine, Ghent University, Ghent, Belgium; 26 CRRAG research group, Division of Radiography, Department of Allied Health, School of Health and Medical Sciences, City St George’s, University of London, London, UK; 27 Magnitiki Tomografia Kerkiras, Corfu, Greece

**Keywords:** Artificial intelligence, Diagnostic imaging, Europe, Radiation oncology, Social media

## Abstract

****Objective**:**

Artificial intelligence (AI) is transforming medical imaging and radiation oncology, yet limited understanding and access to education hinder adoption. This study, led by the European Society of Medical Imaging Informatics (EuSoMII) in collaboration with the European Federation of Radiographer Societies (EFRS), aimed to create an accessible, centralised, searchable database including all AI courses in Europe.

****Materials and methods**:**

An electronic survey was developed to collect data on European AI course characteristics, such as format, delivery, content, target audience and European Qualifications Framework (EQF) level. This was disseminated via purposive sampling through social media and mailing lists of the EuSoMII and the EFRS between September 2024 and January 2025. Quantitative data were analysed using descriptive statistics and visual representations using Python Seaborn and Geopandas.

****Results**:**

This study identified 29 AI courses in Europe. Of them, 53.6% were offered by universities. Courses targeted radiographers (59%), medical physicists (52%), and radiologists (41%), mainly at EQF level 7 (44.4%). Most courses were standalone (65.6%) and online (55.1%), while 41.3% were free of charge. English was the primary language of delivery (79%).

****Conclusions**:**

Different AI courses across Europe offer some entry-level knowledge but are often short in duration. Expanding formats, building practical competencies, providing multilingual access, and European-wide reach are essential for meaningful, practical, and equitable AI integration.

****Relevance statement**:**

With the scaling-up of AI adoption in medical imaging and radiation oncology, there is a variety of AI education provisions currently available. Accessing these options via an open, centralised, regularly updated database enables people to make an informed decision about their training and practise safely and meaningfully.

**Key Points:**

- We identified 29 different AI European courses varying in language, content, and delivery.
- Many clinical practitioners and researchers are unaware of these resources.
- We need a centralised database for customising AI learning choices and guiding future course design.

**Graphical Abstract:**

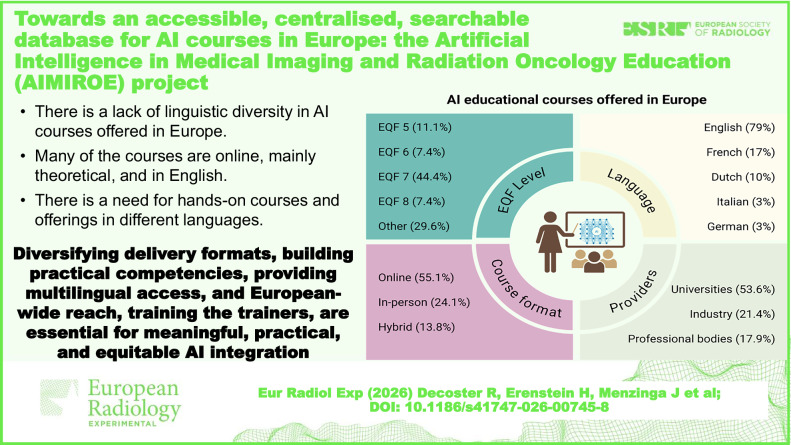

## Background

Recent advances in computational power, infrastructure, and algorithmic design have significantly accelerated the development and application of artificial intelligence (AI) in radiology clinical practice [1–5]. Intelligent systems are now integrated into scheduling and planning workflows, enabling more efficient organisation of diagnostic imaging departments and optimal allocation of human resources [6]. These systems also support radiologists, radiographers, and other medical imaging and radiation oncology (MIRO) professionals in clinical decision-making, contributing to improved quality and consistency in diagnostic procedures [4–7], and more efficient treatment planning, outcome prediction, and workflow optimisation in radiation oncology procedures [8].

While these developments are mainly promising, they also introduce new challenges, risks and potential errors [3]. The opacity and complexity of AI systems raise concerns about transparency, accountability, and informed decision-making [2, 4], requiring robust governance to be in place [9]. These new challenges also underscore the importance of AI education for equipping MIRO professionals with a foundational understanding of AI [5, 10, 11]. Without this knowledge, skills, and competencies, professionals may struggle to embrace AI applications for safe and effective clinical use, be unable to recognise and monitor AI-generated errors, or find it hard to critically assess AI outputs and explain them to patients; these can potentially compromise trust and clinical safety [3]. Successful and ethical implementation of AI in healthcare requires targeted education and training [2, 5, 12]. MIRO clinical staff must be equipped with the necessary knowledge and skills to understand, evaluate, and responsibly use AI technologies in clinical practice [9, 13].

The European Union (EU) has taken a proactive role in regulating AI through the introduction of the AI Act [14]. A key requirement is that AI-generated outputs must be explainable. Understanding AI training and testing is crucial for identifying potential biases or underrepresentation of specific populations, which could lead to inaccurate or even harmful outcomes [9, 15]. Healthcare institutions will be legally required to enhance their employees’ digital literacy and understanding of AI technologies. However, keeping this up-to-date presents a significant challenge in the field of AI that is rapidly evolving [16, 17].

MIRO professionals and other healthcare practitioners play a critical role in safely and effectively integrating AI into clinical practice [18]. As AI becomes more embedded in clinical practice, the demand for structured, accessible, and up-to-date educational programmes grows. Understanding AI tools requires a combination of technical knowledge, clinical expertise, ethical awareness, and communication skills [7, 19, 20]. To ensure effective and sustainable learning for both current and future MIRO professionals, a tiered educational approach, adapted to varying levels of complexity and specific professional roles, may be essential. To support increasing AI literacy amongst MIRO professionals, different AI courses have started to emerge, but they are not always accessible, address the same audience or provide the same learning objectives.

There is a pressing need for a comprehensive, centralised, searchable database of AI-related educational programmes tailored to MIRO professionals at various educational levels, languages, and delivery formats. Such a resource would ease access to training opportunities, promote transparency and standardisation of learning resources, and help identify gaps in current offerings. It would also support the development of digital literacy across the medical imaging professions, ensuring that practitioners are prepared to navigate the evolving landscape of AI in healthcare. By mapping existing educational resources, this study aims to contribute to the development of a more inclusive, equitable and accessible AI education database in MIRO.

## Methods

### Study design

This is a cross-sectional study employing an online survey as the data collection instrument. The online survey was selected as an efficient, inexpensive and well-tested data collection method, allowing for the reliable collection of a sufficient number of both qualitative and quantitative data to thoroughly address a research aim [21], while also offering the advantage of studying diverse populations [22]. Digital transformation and the ongoing increase in social media popularity have also contributed to increased respondent engagement and swift data analysis [23]. Reporting of this study’s findings aligns with both the (a) Improving the Quality of Web Surveys: The Checklist for Reporting Results of Internet E-Surveys (CHERRIES) [24] and (b) Strengthening the Reporting of Observational Studies in Epidemiology (STROBE) guidelines [25].

### Ethics

Ethical approval was sought and received by the Sociaal-Maatschappelijke Ethische Commissie (SMEC) at Katholieke Universiteit Leuven (Ref: G-2024 08 2214). Informed consent was obtained from all participants using a dedicated tick box on the first page of the survey. In addition, all participants were well informed regarding the aim of the study, the anticipated time for survey completion, the type of data to be stored, data storage policies, and contact details of the principal investigator. Participation was voluntary, and no incentives were offered to the respondents.

### Survey design

An open online survey designed on Qualtrics [26] was used to collect data for this study. The survey was designed based on team discussions and expert input, and it was piloted with seven medical imaging researchers to ensure content and face validity [27]. The final survey instrument, consisting of 20 pages, included 22 questions, five of which were closed-ended, two were open-ended, and 15 were multiple-choice. This allowed for free-text responses to include all eventualities. Adaptive questioning was employed, where sensible, to reduce the number and complexity of questions. Also, respondents were able to navigate to previous questions using a dedicated button, in case they wished to change their responses. Duplicate entries were prevented by monitoring the Internet Protocol (IP) addresses of the client computers. However, anonymity of the responses was maintained through a built-in system that flags duplicate responses without revealing the IP address to the researchers. Finally, all AI course characteristics were self-reported by the respondents and not assigned by the research team. The entire survey can be found as Supplementary material.

### Survey dissemination

The survey was disseminated using purposive sampling via email to the professional networks of the research team and snowball sampling via social media by the key study’s endorsing bodies: European Society of Medical Imaging Informatics (EuSOMII), European Federation of Radiographer Societies (EFRS), and European Society of Radiology (ESR). The survey remained active from September 9, 2024, to January 9, 2025, to increase participation. Monthly reminders were sent by the research team and endorsing societies, either via email or social media posts.

### Participants

Participants were invited to complete the survey if they were a MIRO professional (*e.g.*, radiologist, radiographer, radiation oncologist, medical physicist, technical physician, engineer, computer scientist, etc), working in Europe, and were aware of an AI-related course in their field that they had attended or taught at. Vendor representatives could also participate in this survey if they fulfilled the above eligibility criteria.

### Data analysis

The survey was initially cleared of all invalid and incomplete responses. Only responses that had been at least 75% complete on Qualtrics were included in analysis, because they were offering enough data to meaningfully populate the educational AI database. Quantitative data were analysed using descriptive statistics and visual representations of the responses using Python Seaborn and Geopandas [28–30]. All responses were processed into a summative table, whilst full responses are disseminated through the open online database [31]. All free-text responses were assessed by two researchers and assigned to categories with common content, where relevant. Any duplicate courses were removed, but information on their design or content was merged where available. It must be noted that not all percentages reported in this study sum up to 100%, due to the multiple-choice nature of some survey questions.

## Results

The complete database of AI courses for medical imaging and oncology professionals in Europe can be found on the following webpage: https://www.eusomii.org/courses/ [31] (At the time of publication, the webpage is still under construction, as we check the accuracy and completeness of all information provided. This database will be regularly updated with more data entries, as they become available, with allocated funding approved by the EuSoMII Executive Board).

### Response characteristics

**Fig. 1:**
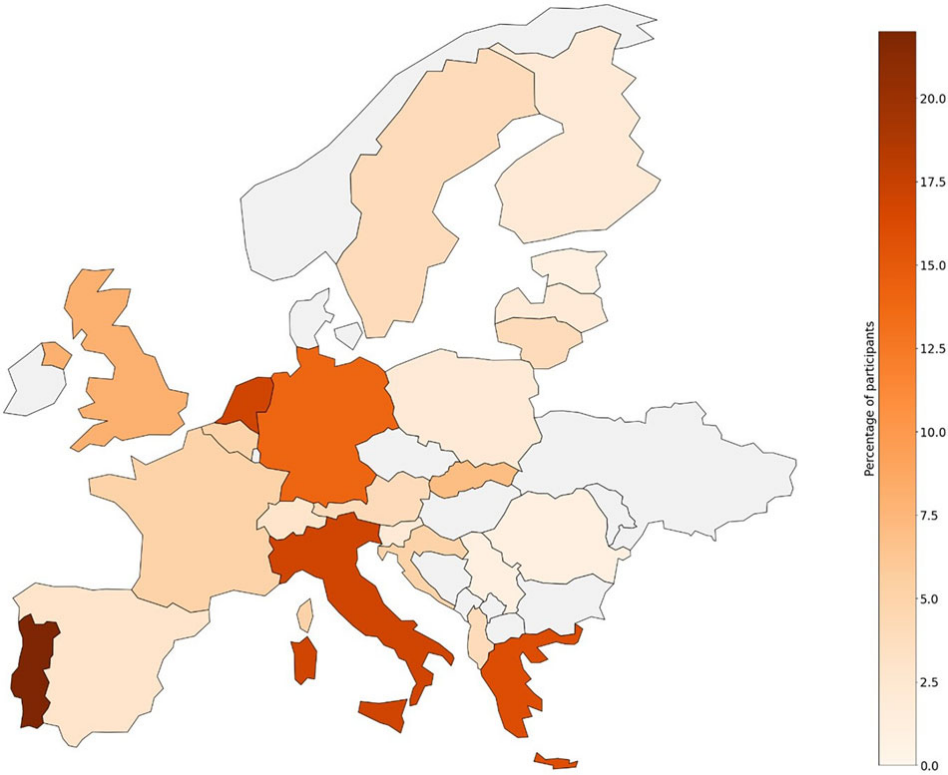
Distribution of survey respondents across Europe. Note the denser concentration of respondents in Western Europe

A total of 215 responses were collected and filtered to exclude incomplete and duplicate entries. The final number of complete responses resulted in the identification of 29 unique AI courses. Participants came from across Europe, with a notable concentration in Western Europe (Fig. 1).

**Fig. 2:**
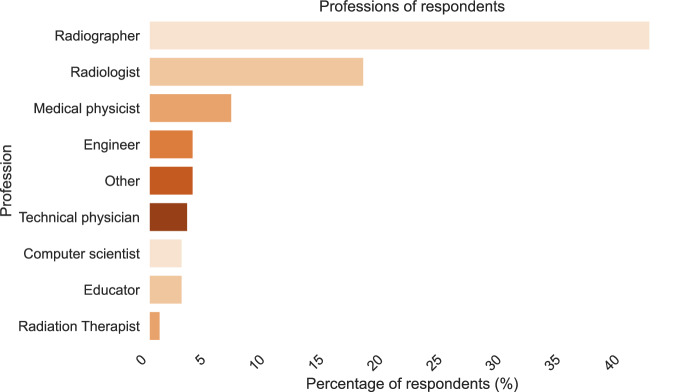
Professional background distribution of survey respondents

Approximately 60% of respondents were radiographers or radiologists. About 17% identified with more technically oriented roles, such as medical physicists, engineers, technical physicians (a hybrid technical-medical role introduced in the Netherlands to bridge technology and patient care), or computer scientists (Fig. 2).

### General course characteristics

Of the 29 courses, 15 (51.7%) were offered by universities or other educational institutions, followed by industry (6, 20.1%) and professional bodies (5, 17.2%). The remaining 3 courses (10.3%) were provided by research institutions or other organisations. The intended audiences varied; numerous courses were reported to be relevant for all professionals within the medical imaging AI ecosystem (Table 1). Of those professions specifically mentioned, radiographers, medical physicists and radiologists were mentioned as a potential audience in 40–60% of the reported courses (Reported educational levels, as defined by the European Qualifications Framework (EQF) [32], varied; however, 12 courses (41.4%) were reported to be EQF Level 7 (postgraduate level/master’s level). Other or non-classified levels were also reported (10, 34.4%), whilst the remainder were covered by EQF levels 5 (vocational training or equivalent), 6 (bachelor’s degree or equivalent) and 8 (doctorate or equivalent), as shown in Table 2.

**Table 1:** Intended audience of different AI courses in this database

Profession	Courses	
	Number	(%)
Radiographers	17	58.6
Medical physicists	15	51.7
Radiologists	12	41.3
Computer scientists	8	27.6
Technical physicians	8	27.6
Radiation Oncologists	8	27.6
Engineers	8	27.6

**Table 2:** EQF levels of reported AI courses

EQF level	Courses	
	Number	(%)
EQF5	3	10.3
EQF6	2	7.4
EQF7	12	41.4
EQF8	2	6.9
Other	10	34.4

Standalone courses, which are independent courses not necessarily part of a larger educational programme, were most frequently reported (19, 65.5%), followed by those forming part of an educational programme (6, 20.1%). In 14 reported courses (48.3%), the duration was a week or less (Table 3). Additionally, the respondents often noted that a course could be taken at one’s own pace due to offline asynchronous e-learning approaches.

**Table 3:** Duration of reported courses

Duration	Courses	
	Number	(%)
1 day	4	13.8
2–3 days	7	24.1
1 week	3	10.3
1 month	2	6.9
1 year	2	6.9
Longer than 1 year	3	10.3
Other	8	27.6

The majority of the courses (16, 55.1%) were designed to be delivered entirely online, either via classes or individual e-learning (Table 4). Another 8 courses (27.6%) were meant to be delivered in person, and only 4 courses (13.8%) had a hybrid approach. In 12 provisions (41.3%), there were no associated costs with the course, indicating a significant number of cost-free courses. Regarding the cost of fee-paying courses, the mean cost was 2,088 euros. The median cost was 495 euros, with a range between 171 and 9628 euros, with duration, delivery format, and number of educational hours ranging accordingly.

**Table 4:** Mode of delivery for reported courses

Means of delivery	Courses	
	Number	(%)
Online classes	11	37.9
In person	8	27.6
Online individual e-learning	5	17.2
Hybrid	4	13.8
Missing	1	3.4

Most AI courses (22, 75.9%) were accessible to those outside of the related academic institution, with the remaining 7 (24.1%) being exclusively offered only to those linked to specific programmes or society memberships. In 12 courses (41.4%) no formal academic credits were offered. English was the most common language (25, 86.2%) used for AI courses in Europe (Table 5). Six of the courses (20.1%) were offered in more than one language, *e.g.*, English, Dutch and French, while 11 courses (37.9%) were taught in languages other than English.

**Table 5:** Language of delivery for reported courses

Language	Courses	
	Number	(%)
English	25	86.2
French	6	20.7
Dutch	3	10.3
Italian	1	3.4
German	1	3.4

The majority of the educational curriculum delivered during the courses related to clinical AI applications (25, 86.2%), AI basic principles (23, 79.3%), and AI terminology (20, 69%). Less frequently addressed areas included programming (9, 31%), quality assurance (9, 31%), and implementation theories (5, 17.2%) (Table 6).

**Table 6:** Curriculum content of different AI courses in the database

Topic	Courses	
	Number	(%)
Clinical AI applications	25	86.2
AI basic principles	23	79.3
AI terminology	20	69
Impact of AI on the profession	18	62.1
AI implementation	18	62.1
Ethical challenges	16	55.2
AI research	15	51.7
Regulatory/legal challenges	15	51.7
AI development	14	48.3
Societal challenges of AI	12	41.4
Health technology assessment	12	41.4
Impact on patient management	11	37.9
Quality assurance	9	31
Programming	9	31
Implementation theories	5	17.2

## Discussion

AI becomes increasingly integrated into healthcare systems; the need for structured, formalised education among medical imaging professionals has become both urgent and essential [3]. This study aimed to assess the availability and diversity of AI-related educational resources for medical imaging professionals across Europe.

The findings of this study suggest that most AI courses in this database are brief (nearly half of these courses last 1 week or less) when teaching hours or academic credits are considered. Also, most courses were delivered online; online learning strategies may be beneficial because they enhance accessibility. On the contrary, online formats may not always enable hands-on educational activities, although some online formats allow for interactive learning, simulations, and case-based learning. Such concerns have already been stressed in the literature, highlighting that short educational courses might limit learning quality and the acquisition of transferable knowledge and skills [33]. Hybrid approaches, employing blended learning, as well as efficient self-directed learning strategies, could be considered to balance the advantages and disadvantages of different modes of delivery [34, 35]. Most of the reported courses were offered at EQF Level 7, indicating a formal accreditation system with pedagogical input from higher education institutions (HEIs). There is a clear role and need for HEIs to work on expanding and diversifying AI education across all educational levels and on scaling up knowledge, skills and competencies to support the workforce and accelerate implementation. This is further strengthened by recent research confirming a general lack of AI educational provisions at the undergraduate level, both for medical students and radiographers [36, 37]. Furthermore, blueprints for AI-related digital competencies for MIRO professionals are still lacking; as of October 2025, the EFRS Imaging Informatics working group has initiated the development of an AI educational framework for radiographers across Europe.

Several courses lacked academic accreditation, which might act as a counter incentive for clinical practitioners, given fitness for practice requirements in different countries [38, 39]. Professional bodies worldwide, such as the ESR, the United Kingdom Society and College of Radiographers, and the American Society of Radiologic Technologists, have suggested AI education content for their members [7, 40].

A significant proportion of the responses originated from Western Europe, highlighting potential regional disparities in AI education and awareness. This could reflect either a genuine gap in educational provisions or a limited reach of the ESR, EuSoMII, and EFRS networks in other parts of Europe, potentially due to English language being the predominant language used in communication with the membership. This finding aligns with recent research highlighting the generally limited availability of AI resources for radiographers across Europe [11], while a recent survey among ESR’s members indicated that conferences were the preferred source of AI-related learning [41], further confirming the relative lack of more formalised, University-led educational provisions on AI.

The limited linguistic diversity of courses, predominantly offered in English, may further restrict accessibility for non-English-speaking professionals. It has been stressed that non-English professionals might miss important educational opportunities due to language barriers [42]. This, in turn, might prevent the use of AI applications and minimise the efficacy of AI adoption [43]. The development of a centralised course database aims to provide ongoing accessibility and transparency of current and future AI educational provisions. It might also encourage colleagues from low- or middle-income countries to engage with those AI courses that are more accessible to them (language, cost, mode of delivery), but also highlight to educators and educational researchers the obvious gaps in content, language or delivery format, that need to be further developed or explored. Overall, the hope is that this database will serve as a reference point to promote any continuous professional development or related courses to potential learners.

MIRO professionals operate at the critical interface between patient care and technological innovation [44], being in a unique position to guide the ethical and sustainable adoption of AI in clinical practice [16]. Recent legal frameworks, such as the EU AI Act [13], and professional standards like those set by the Health and Care Professions Council [33], now mandate digital competencies and AI literacy, reinforcing the necessity of well-structured and accessible foundational AI training. Professionals of different seniority or roles will naturally have diverse educational needs, and this diversity should be reflected in future AI educational provisions in a tiered educational approach: foundational AI literacy for all professionals, and advanced competencies for those in leadership or strategic implementation roles, who will be acting as AI ambassadors in clinical or academic/research settings [36, 45].

This necessity reflects a broader ethical requirement: MIRO professionals must continue to deliver safe, high-quality, patient-centred care, while also understanding and managing increasingly complex technologies [16]. For instance, the opacity of many AI systems raises concerns about transparency, accountability, and informed decision-making [46–48]. The so-called ‘black box’ effect of AI, a phenomenon associated with inherently opaque AI algorithms, can prevent professionals without proper, in-depth education from detecting and escalating AI-induced errors, while also compromising patient trust in these technologies [49]. Post-market surveillance of AI tools is paramount to ensure stability of performance and safety over time [50]. This is strengthened by recent research showing notable variability among different large language models used in radiology, highlighting the key role of expert human oversight after AI deployment [51]. In addition, large language models demonstrated certain limitations in justifying CT referrals [52], further supporting the need for optimal AI education and literacy to maintain oversight and manage quality assurance over AI tools. Without adequate training, clinical practitioners may be unable to critically assess AI outputs or communicate their implications to patients, potentially undermining clinical safety and public trust [9]. Beyond AI performance considerations, AI research and innovation need to adhere to standardised protocols, robust methodologies, and transparency [53, 54]. The above evidence supports the need for accessible, harmonised, standardised approaches in AI to safely integrate it into clinical practice. The development of the AIMIROE database resonates with the above principles by offering an accessible, centralised, and searchable platform for AI educational initiatives in Europe.

It is essential to integrate novel knowledge, skills, and competencies into AI education frameworks that will shape future academic curricula at EQF levels 5 to 7. The newly formed EFRS Imaging Informatics Working Group, led by many of the coauthors of this study, has already started to work on AI education benchmarking for radiographers. This work will result in the development of a “scope of practice” for radiographers in imaging informatics, identification of the knowledge, skills, and competencies needed for future professionals, and alignment of the above with EQF levels 6 and 7. This integration will ensure that future professionals are well equipped to effectively use AI tools, understand their limitations and ethical implications, and mitigate them as required. It will also empower them to lead in patient care and drive digital transformation [36]. Importantly, the need for robust AI knowledge extends beyond current and future healthcare professionals. AI educators must also possess a substantial level of understanding to effectively guide learners and uphold the quality of AI education. Previous research indicates that clinical and academic educators often lack the necessary skills to effectively teach about and with AI [19]. Academic institutions should, therefore, invest in ‘’training the trainers” and provide the necessary support, funding, and time needed to develop, deploy and evaluate AI educational tools [55–58].

This study has several limitations. First, although English is widely recognised as the *lingua franca* across Europe, the use of the English language in the survey dissemination might have resulted in linguistic bias. In addition, the geographic concentration of the responses in Western Europe may not accurately reflect the broader European context. The number of AI courses identified is relatively small (29 unique courses), which might limit the generalisability of the findings, but regular updates will ensure their currency and completeness in time. Moreover, the use of purposive sampling, although justified when the focus is on information-rich cases relative to the research question and the efficient use of limited resources [59], might have resulted in selection bias in this study. Furthermore, the course characteristics (*e.g.*, EQF level, delivery format, cost, etc.) were self-reported and not externally validated by the research team. Additionally, professional representation is skewed, with a predominance of radiographers and limited input from other medical imaging subfields, such as radiation oncology. This difference may be partially attributed to the limited reach of this survey or the actual representation of these professions within the MIRO ecosystem in Europe [60, 61]. Future database updates will be targeted at increasing the pool of professionals and countries represented. An updated survey for this database is planned for 2026 to ensure these gaps are addressed.

In conclusion, we identified 29 different AI-related educational opportunities for medical imaging and oncology professionals in Europe, increasingly delivered through online platforms, often at a postgraduate level. However, accessibility remains uneven, geographically and linguistically. To ensure the responsible and effective integration of AI into healthcare, it is essential to embed AI education into formal curricula, support the trainers, and promote equitable access across all regions and professional groups. The findings underscore a shared responsibility among higher education institutions, policymakers, and professionals to foster a sustainable and ethical AI ecosystem in healthcare through accessible AI education for all.

## Supplementary information

ELECTRONIC SUPPLEMENTARY MATERIAL

## Data Availability

The dataset generated and analysed during the current study is available from the corresponding author on reasonable request.

## Abbreviations

AI: Artificial intelligence
EFRS: European Federation of Radiographer Societies
EQF: European Qualifications Framework
ESR: European Society of Radiology
EU: European Union
EuSoMI: European Society of Medical Imaging Informatics
MIRO: Medical imaging and radiation oncology
