# Supplementary material for: Towards an accessible, centralised, searchable database for AI courses in Europe: the Artificial Intelligence in Medical Imaging and Radiation Oncology Education (AIMIROE) project
Source: Eur Radiol Exp. 2026 May 29;10:80. doi: 10.1186/s41747-026-00745-8 (PMC13221536; doi:10.1186/s41747-026-00745-8)
Supplement: Supplementary file 1 — ELECTRONIC SUPPLEMENTARY MATERIAL [file 41747_2026_745_MOESM1_ESM.pdf]

Towards an accessible, centralised, searchable database for AI courses in Europe: the Artificial Intelligence in Medical Imaging and Radiation Oncology Education (AIMIROE) project

Eur Radiol Exp (2026) Decoster R, Erenstein H, Menzinga J, et al.

ELECTRONIC SUPPLEMENTARY MATERIAL**Text of the survey**

1. Which country are you based at?

- ◯ Albania
- ◯ Andorra
- ◯ Austria
- ◯ Belarus
- ◯ Belgium
- ◯ Bosnia and Herzegovina
- ◯ Bulgaria
- ◯ Croatia
- ◯ Cyprus
- ◯ Czech Republic
- ◯ Denmark
- ◯ Estonia
- ◯ Faroe Islands
- ◯ Finland
- ◯ France
- ◯ Germany
- ◯ Gibraltar
- ◯ Greece
- ◯ Greenland
- ◯ Hungary
- ◯ Iceland
- ◯ Ireland

- Italy
- Kosovo
- Latvia
- Liechtenstein
- Lithuania
- Luxemburg
- Malta
- Moldova
- Monaco
- Montenegro
- Netherlands
- North Macedonia
- Norway
- Poland
- Portugal
- Romania
- Russia
- San Marino
- Serbia
- Slovakia
- Slovenia
- Spain
- Sweden
- Switzerland

- Turkey
- Ukraine
- UK
- Vatican City

2. What is the type of the organisation you are employed by?

- [ ] Hospital
- [ ] Industry
- [ ] Professional body
- [ ] University
- [ ] Other. Please explain: ______________________________

3. What is your professional background?

- [ ] Radiologist
- [ ] Radiographer
- [ ] Oncologist
- [ ] Medical physicist
- [ ] Technical physician
- [ ] Computer scientist
- [ ] Engineer
- [ ] Other. Please explain: ______________________________

4. Please name your AI course (also add the weblink, if known and available)

____

5. Is you course delivered (please choose below) by:

○ A University/other educational institution. Please explain:____

○ A research institute. Please explain:____

○ A professional body. Please explain:____

○ A company/industry. Please explain:____

○ Other organisation. Please explain:____

6. Please select the EQF (European Qualifications Framework) educational level related to your AI programme:

○ EQF5 (associate degree or equivalent)

○ EQF6 (BSc degree or equivalent)

○ EQF7 (Master's degree or equivalent)

○ EQF8 (Doctorate or equivalent)

○ Other. Please explain: ____

7. Please select if your AI education is part of an educational programme or standalone course:

○ It is part of an educational programme

○ It is a standalone course

○ Other. Please explain: ____

8. Please select what is the intended audience of your AI course (multiple choices feasible):

- ☐ Radiologists
- ☐ Radiographers
- ☐ Computer scientists
- ☐ Engineers
- ☐ Medical physicists
- ☐ Technical physicians
- ☐ Oncologists
- ☐ Everyone in the AI ecosystem of medical imaging and/or radiotherapy
- ☐ Other. Please explain: ____

9. What is the total duration of this course?

- ◯ 1 day
- ◯ 2-3 days
- ◯ 1 week
- ◯ 1 month
- ◯ 1 year
- ◯ Longer than 1 year. Please explain: ____
- ◯ Other. Please explain: ____

10. How is the course delivered?

- ◯ Online classes
- ◯ Online individual e-learning
- ◯ In person
- ◯ Hybrid
- ◯ Other. Please explain:____

11. Is this programme accessible to those outside of your university/ institution?

- ◯ Yes
- ◯ No
- ◯ Other. Please explain:____

12. What is the language of delivery of this course (multiple choices feasible)?

- ☐ English
- ☐ German
- ☐ Dutch
- ☐ Italian
- ☐ Spanish
- ☐ French
- ☐ Other. Please explain: ____________________

13. If the course carries academic credits, how many ECTS credits does it award?

- ○ It does not carry academic credits
- ○ It carries academic ECTS credits. Please explain how many in the box provided: ____________________
- ○ Other. Please explain: ____________________

14. Which organisation is your course accredited by?

- [ ] ESR
- [ ] EuSoMII
- [ ] EFRS
- [ ] EFoMP
- [ ] EANM
- [ ] ESTRO
- [ ] EuSOBI
- [ ] A University. Please explain: ______________________________
- [ ] A regulatory organisation. Please explain: ______________________________
- [ ] Other organisation. Please explain: ______________________________
- [ ] No accredited course
- [ ] I do not know

15. What is the cost of the course?

- ○ No cost associated
- ○ Associated cost is... Please explain: ______________________________
- ○ Other. Please explain: ______________________________

16. Please select the topics/content taught in your AI programme (multiple choices feasible):

- ☐ AI basic principles
- ☐ AI terminology
- ☐ Health technology assessment
- ☐ Ethical challenges
- ☐ Clinical AI applications
- ☐ AI implementation, current and future
- ☐ Regulatory/legal challenges
- ☐ Impact of AI on the profession
- ☐ Societal challenges of AI
- ☐ AI development
- ☐ Impact on patient management
- ☐ Quality assurance
- ☐ AI research
- ☐ Programming
- ☐ Implementation theories
- ☐ Other. Please explain: ______________________________

17. Which learning outcomes are linked with this AI programme?

____

18. Are you the (co-) owner of this course?

◯ Yes

◯ No

◯ Other. Please explain: ____

Display this question:

If 18.Are you the (co-) owner of this course? = Yes

Or 18.Are you the (co-) owner of this course? = Other. Please explain:

19. Are you willing to collaborate with others to expand the reach and audience of your course?

◯ Yes

◯ No

◯ Other. Please explain: ____

20. Are we allowed to contact you for additional information if needed?

◯ No, thanks

◯ Yes (please provide the your contact email below):

____
